# Assessment of reference gene stability in *Rice stripe virus* and *Rice black streaked dwarf virus* infection rice by quantitative Real-time PCR

**DOI:** 10.1186/s12985-015-0405-2

**Published:** 2015-10-24

**Authors:** Peng Fang, Rongfei Lu, Feng Sun, Ying Lan, Wenbiao Shen, Linlin Du, Yijun Zhou, Tong Zhou

**Affiliations:** Institute of Plant Protection, Jiangsu Academy of Agricultural Sciences, Nanjing, 210014 China; Scientific Observing and Experimental Station of Crop Pests in Nanjing, Ministry of Agriculture, China, Nanjing, 210014 China; College of Life Sciences, Nanjing Agricultural University, Nanjing, 210095 China

**Keywords:** Gene expression, RT-qPCR, Reference genes, RSV- and RBSDV-infected rice

## Abstract

**Background:**

Stably expressed reference gene(s) normalization is important for the understanding of gene expression patterns by quantitative Real-time PCR (RT-qPCR), particularly for *Rice stripe virus* (RSV) and *Rice black streaked dwarf virus* (RBSDV) that caused seriously damage on rice plants in China and Southeast Asia.

**Methods:**

The expression of fourteen common used reference genes of Oryza sativa L. were evaluated by RT-qPCR in RSV and RBSDV infected rice plants. Suitable normalization reference gene(s) were identified by geNorm and NormFinder algorithms.

**Results:**

*UBQ 10* + *GAPDH* and *UBC* + *Actin1* were identified as suitable reference genes for RT-qPCR normalization under RSV and RBSDV infection, respectively. When using multiple reference genes, the expression patterns of *OsPRIb* and *OsWRKY*, two virus resistance genes, were approximately similar with that reported previously. Comparatively, by using single reference gene (*TIP41-Like*), a weaker inducible response was observed.

**Conclusions:**

We proposed that the combination of two reference genes could obtain more accurate and reliable normalization of RT-qPCR results in RSV- and RBSDV-infected plants. This work therefore sheds light on establishing a standardized RT-qPCR procedure in RSV- and RBSDV-infected rice plants, and might serve as an important point for discovering complex regulatory networks and identifying genes relevant to biological processes or implicated in virus.

**Electronic supplementary material:**

The online version of this article (doi:10.1186/s12985-015-0405-2) contains supplementary material, which is available to authorized users.

## Background

Rice viral diseases are major threats to rice production and have been distributed worldwide across regions depending on rice cultivation [1]. Two of the most prevalent rice viruses are RSV and RBSDV, which were transmitted by a small brown planthopper (SBPH, *Laodelphax striatellus Fallen*) [2–4]. When infected with RSV at the seedling stage, normally, rice plants grow poorly and often develop folded and twisted leaves, with the central leaves yellowing and withering; and plant growth may terminate and ultimately the plant will die [5–7]. In China, rice stripe is very serious, especially in Jiangsu province, where about 0.6 M ha per year of rice were infected by RSV during the period of 2000 to 2003, increasing to 1 M ha in 2004. In heavily infected fields, rice yield is reduced by 30–50 %, and in some of the most severely infected fields, no harvest is possible [8]. In RBSDV infected rice always develops stunted stems, dark green, twisted leaves, and white waxy swellings along veins on the abaxial surface of the leaves [3, 9, 10]. The disease caused severely damage on rice in most parts of eastern China with due to widespread release of susceptible cultivars. Since the infection damage was very severe in China and Southeast Asia, the understanding of the responses of rice to viral infection, especially gene expression analysis, is very important for developing strategies for disease control [8, 11].

The widely used method to measure transcript abundance is RT-qPCR compared to reverse transcription-polymerase chain reaction (RT-PCR) and northern blot [12–14]. Besides being a powerful tool, RT-qPCR suffers from certain pitfalls, most important being the normalization with a reference gene [15–17]. In recent years, the reference genes, such as those encoding actin (*Actin*), tubulin (*TUB*), glyceraldehyde-3-phosphate dehydrogenase (*GAPDH*), and *18S rRNA*, are often separately chosen for the normalization in RT-qPCR because of their constant expression levels in living organisms [16]. Nevertheless, different studies sometimes proved different or even opposing results of these reference genes, and it was demonstrated that the transcript levels of these genes actually vary under different experimental conditions [18–21]. For example, different expression levels normalized by a different reference gene could be approximately 100 folds [22]. Furthermore, *Myzus persicae’s* actin and GAPDH protein were found to interaction with *Beet western yellows virus* in vitro [23] and some *A.pisumwere’s* genes (*Actin* and *GAPDH*) considered to be potentially related to the transmission of *Peaenation mosaic virus* and *Soybean dwarf virus* [24]. Thus, it is important and necessary to select suitable reference gene(s) for different experimental paradigms, particularly in RSV and RBSDV infection conditions which those appropriate internal reference(s) were not identified [25–27].

In this study, we reported the validation of reference genes to identify the most suitable internal control gene(s) for the normalization of RT-qPCR data upon viral infection in rice plants. Using statistical algorithms geNorm and Norm Finder [28, 29], the stability of 14 candidate reference genes (*Actin*, *UBC*, *18S rRNA*, *EF-1α*, *UBQ 5*, *GAPDH*, *α-TUB*, *β-TUB*, *eIF-4α*, *Actin1*, *UBQ 10*, *TIP41-like*, *EXP* and *Os AOC*) was examined and compared. Two best reference genes were identified more stably expressed than traditional ones in RSV- and RBSDV-infected treatments. Our results further indicated that the combination of these two reference genes provides a good starting point for gene expression analysis in rice viral infection plants by RT-qPCR.

## Results

### Rice infectivity assay

**Fig. 1:**
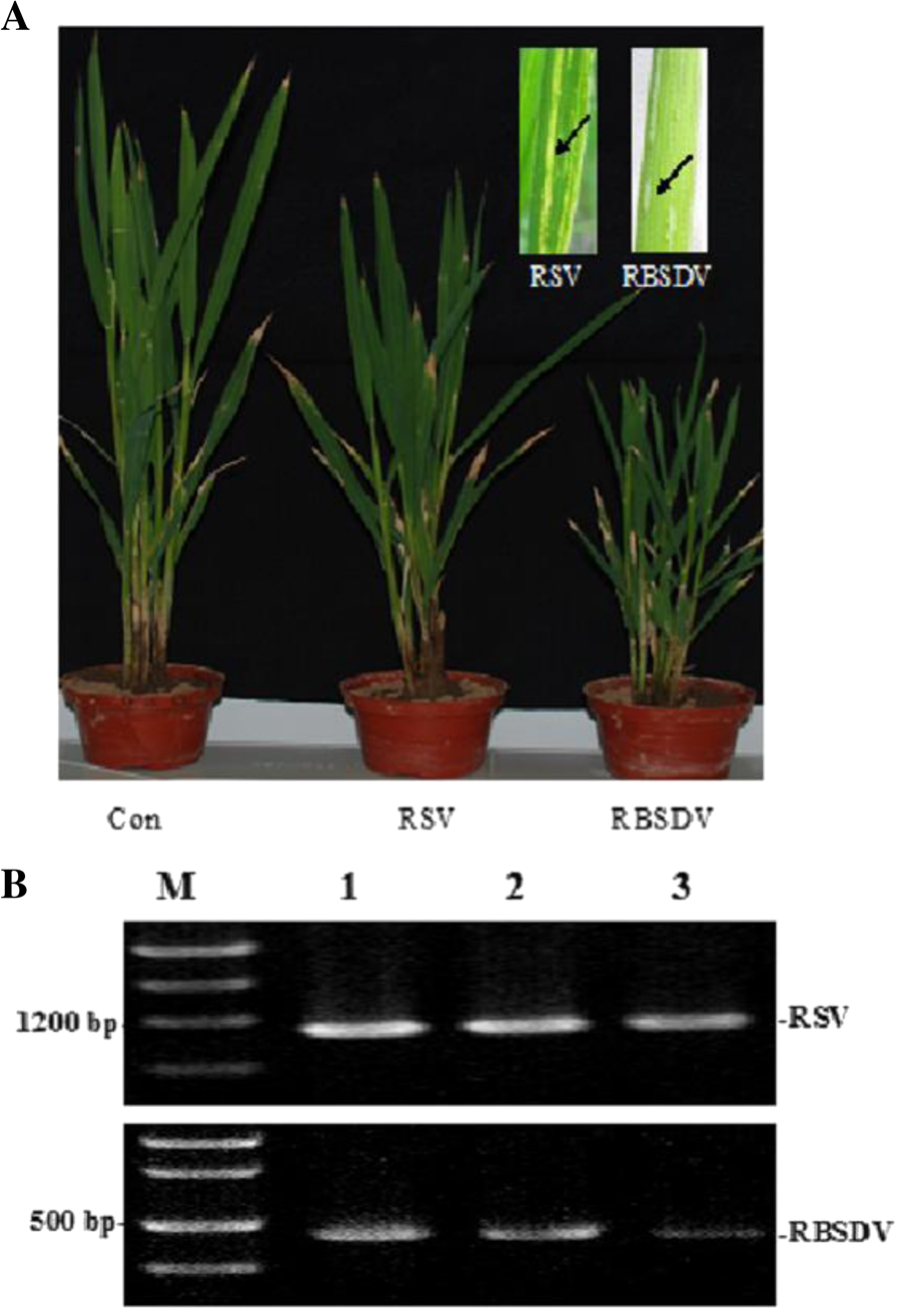
RSV and RBSDV infection in rice plants. **a** Disease symptoms developed on rice during virus infection. From left to right, non-inoculated plant, RSV- and RBSDV-infected rice plants. **b** Agarose gel (2 %) showing RSV and RBSDV in inoculated rice (1–3 means three replications)

We characterized the phenotype of RSV- and RBSDV-infected rice plants, and the symptoms were allowed to develop under the controlled environmental conditions. RSV-infected rice developed folded and twisted leaves, with the central leaves yellowing and withering. Meanwhile in RBSDV-infected rice plants, it developed stunted stems and white waxy swellings along veins on the abaxial surface of the leaves (Fig. 1a). RSV and RBSDV were respectively detected in inoculated rice, using RT-PCR (Fig. 1b).

### Identification of candidate reference genes

**Fig. 2:**
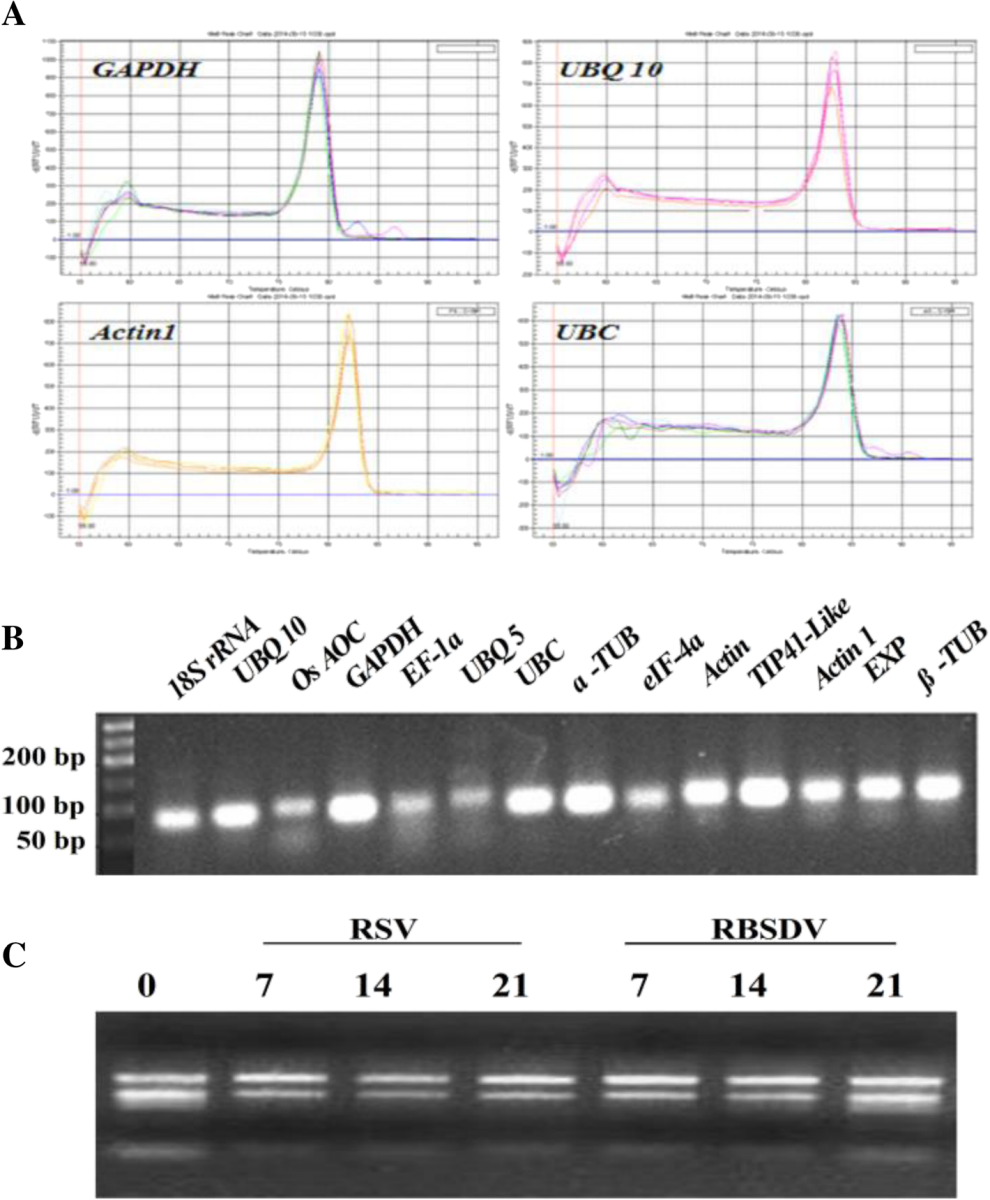
The specificity of real time PCR amplification. **a** Dissociation curves for four representative genes with single peak obtained from three technical replicates. **b** Agarose gel (2 %) showing amplification of a specific PCR product of expected size for each reference gene tested in the study; **c** Agarose gel showing the quality of RNA (0, 7, 14 and 21 means days after RSV- and RBSDV-infection)

In order to evaluate the expression stability of reference genes, the candidates should be identified first. In our test, primers were designed for the fourteen commonly used reference genes, identified by BLASTN and TBLASTN searches at National Center for Biotechnology Information (NCBI). The gene names, accession numbers, gene description and primer sequences were all provided in Table 1. First, melting curve for four representative genes (Dissociation curves for all other genes with single peak were not shown) and agarose gel analysis confirmed that each primer set gave a single amplified product of desired size (Fig. 2a and b). Therefore, these genes were selected for RT-qPCR validation. It is also important to accurately quantitate the quality of RNA before reverse transcription. The concentration and quality of isolated RNA were determined using the NanoDrop 2000 spectrophotometer. Agarose gel electrophoresis assay also confirmed the integrity of RNA samples (Fig. 2c).

**Table 1:** Candidate reference genes and their primer sequences used in this study

Gene name	Accession number	Gene description	Primer sequence (5′ → 3′)	Amplicon length (bp)
*Actin*	AK058421	Actin	F-CAGCCACACTGTCCCCATCTA	86
			R-AGCAAGGTCGAGACGAAGGA	
*UBC*	AK059694	Ubiquitin-conjugating enzyme E2	F-CCGTTTGTAGAGCCATAATTGCA	76
			R-AGGTTGCCTGAGTCACAGTTAAGTG	
*18S rRNA*	AK059783	18S ribosomal RNA	F-CTACGTCCCTGCCCTTTGTACA	65
			R-ACACTTCACCGGACCATTCAA	
*EF-1α*	AK061464	Eukaryotic elongation factor1-alpha	F-TTTCACTCTTGGTGTGAAGCAGAT	103
			R-GACTTCCTTCACGATTTCATCGTAA	
*UBQ 5*	AK061988	Ubiquitin 5	F-ACCACTTCGACCGCCACTACT	69
			R-ACGCCTAAGCCTGCTGGTT	
*GAPDH*	AK064164	Glyceraldehyde-3-Phosphate dehydrogenase	F-AAGCCAGCATCCTATGATCAGATT	79
			R-CGTAACCCAGAATACCCTTGAGTTT	
*α-TUB*	AK067721	Alpha-tubulin	F-GGAAATACATGGCTTGCTGCTT	89
			R-TCTCTTCGTCTTGATGGTTGCA	
*β-TUB*	AK072502	Beta-tubulin	F-GCTGACCACACCTAGCTTTGG	82
			R-AGGGAACCTTAGGCAGCATGT	
*eIF-4α*	AK073620	Eukaryotic-initiation factor 4α	F-TTGTGCTGGATGAAGCTGATG	76
			R-GGAAGGAGCTGGAAGATATCATAGA	
*Actin1*	AK100267	Actin1	F-CTCCCCCATGCTATCCTTCG	67
			R-TGAATGAGTAACCACGCTCCG	
*UBQ 10*	AK101547	Ubiquitin 10	F-TGGTCAGTAATCAGCCAGTTTGG	65
			R-GCACCACAAATACTTGACGAACAG	
*TIP41-Like*	AK103511	TIP41-like family protein	F-GTTTGGATGAACCCCGCAA	75
			R-GGCAACAAGGTCAATCCGATC	
*EXP*	Os06g11070	Expressed protein	F-AGGCTGGTCGAGGAGTCCAT	84
			R-TTCTCCTCCCTAGCGAACACCT	
*Os AOC*	AI493664	Allene oxide cyclase	F-CCACCATCACAGATCGGATCTT	78
			R-GCGGTCAGAGCGAAAGTAGCTA

**Fig. 3:**
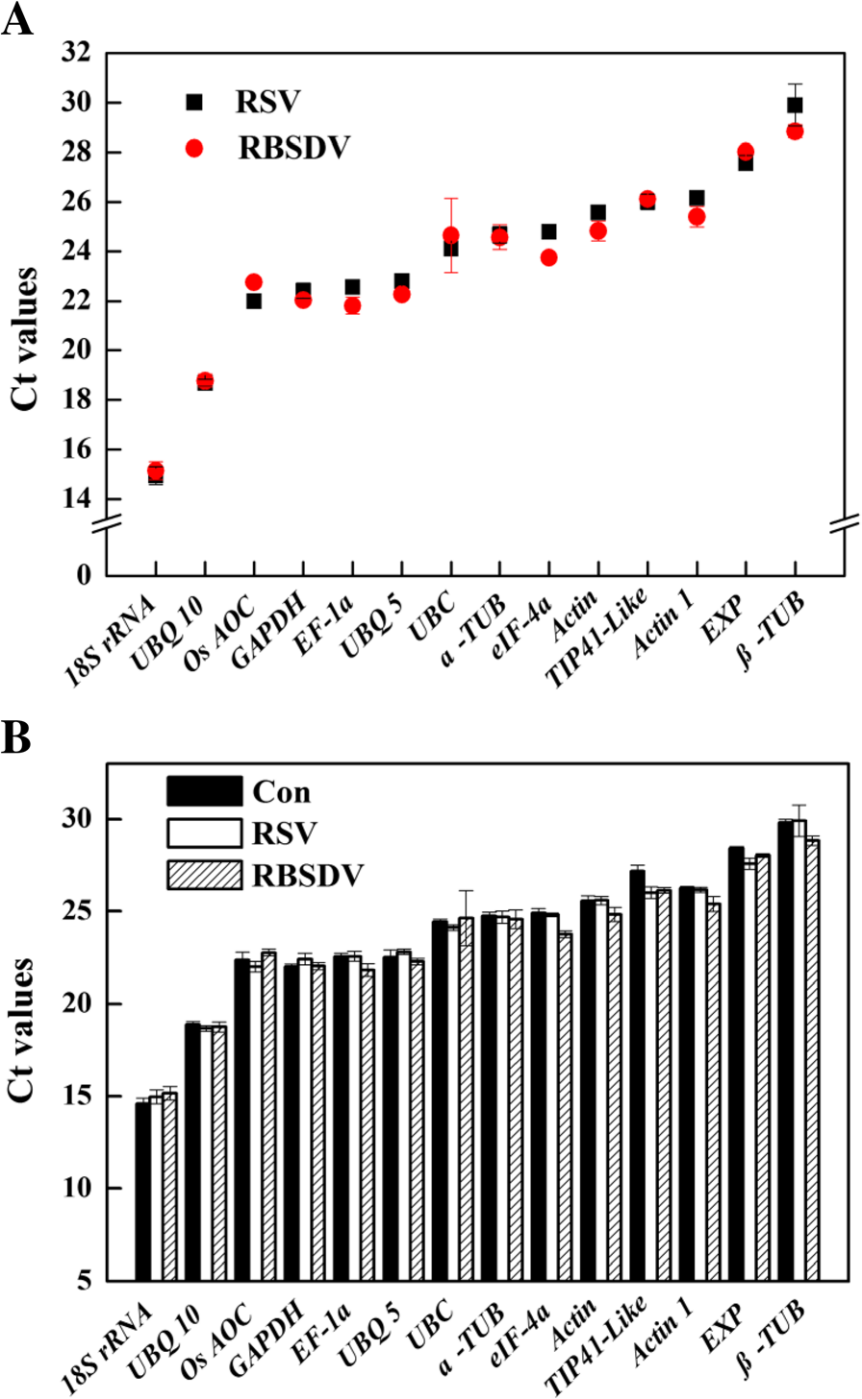
Expression levels of candidate reference genes. **a** Average cycle threshold (Ct) values for the 14 candidate reference genes used in this study; **b** Comparison of the expression levels of candidate reference genes in different treatments (Con: virus-free SBPHs infection; RSV: RSV SBPHs infection; RBSDV: RBSDV SBPHs infection). 14-day-old rice seedlings were inoculated with or without viruliferous nymphs (RSV and RBSDV) for 3 days. Total RNA was extracted from RSV- and RBSDV-infected seedlings, respectively. Values were given in the form of RT-qPCR quantification cycle numbers across all tested samples. Bars indicate standard error of the mean

To give an overview picture of the relative abundance of candidate reference genes, the calculated cycle threshold (Ct) values were determined for each gene across all the tested RSV- and RBSDV-infected samples (Fig. 3a). RT-qPCR analysis indicated that 14 candidate reference genes exhibited different levels of abundance. The Ct values ranged from 14 to 30, with the most lying between 22 and 28. *18S rRNA* and *UBQ 10* were more abundantly transcribed than others, while *β-TUB* was the least expressed gene across all the tested samples.

Because the transcript levels of reference genes actually varied under different experimental conditions [18, 21], we analyzed if virus infection (RSV and RBSDV infection) altered the expression of any of the 14 candidate genes. The Ct values obtained for each gene were compared in RSV- and RBSDV-infected against virus-free treatment. In this study, our results showed that each candidate reference gene expressed constantly in different treatments (Fig. 3b). Variation in transcript quantity of RSV- and RBSDV-infected samples revealed that each tested gene approximately exhibited the similar transcript levels. Therefore, above 14 references genes were selected in the subsequent analyses.

### Comparison of the expression stability of housekeeping genes by geNorm and NormFinder

The expression stability of abovementioned candidate reference genes could be assessed by different software programs. To date, the most popular and useful method is geNorm algorithm [22, 28]. The geNorm is a statistical algorithm which determines the gene stability measure (M) of all the genes under investigation, based on the geometric averaging of multiple control genes and mean pairwise variation of a gene from all other control genes in a given set of samples [28]. It relies on the principle that the expression ratio of two ideal internal control genes is identical in all the samples, regardless of the experimental condition and cell-type. Genes with the lowest M values have the most stable expression.

**Fig. 4:**
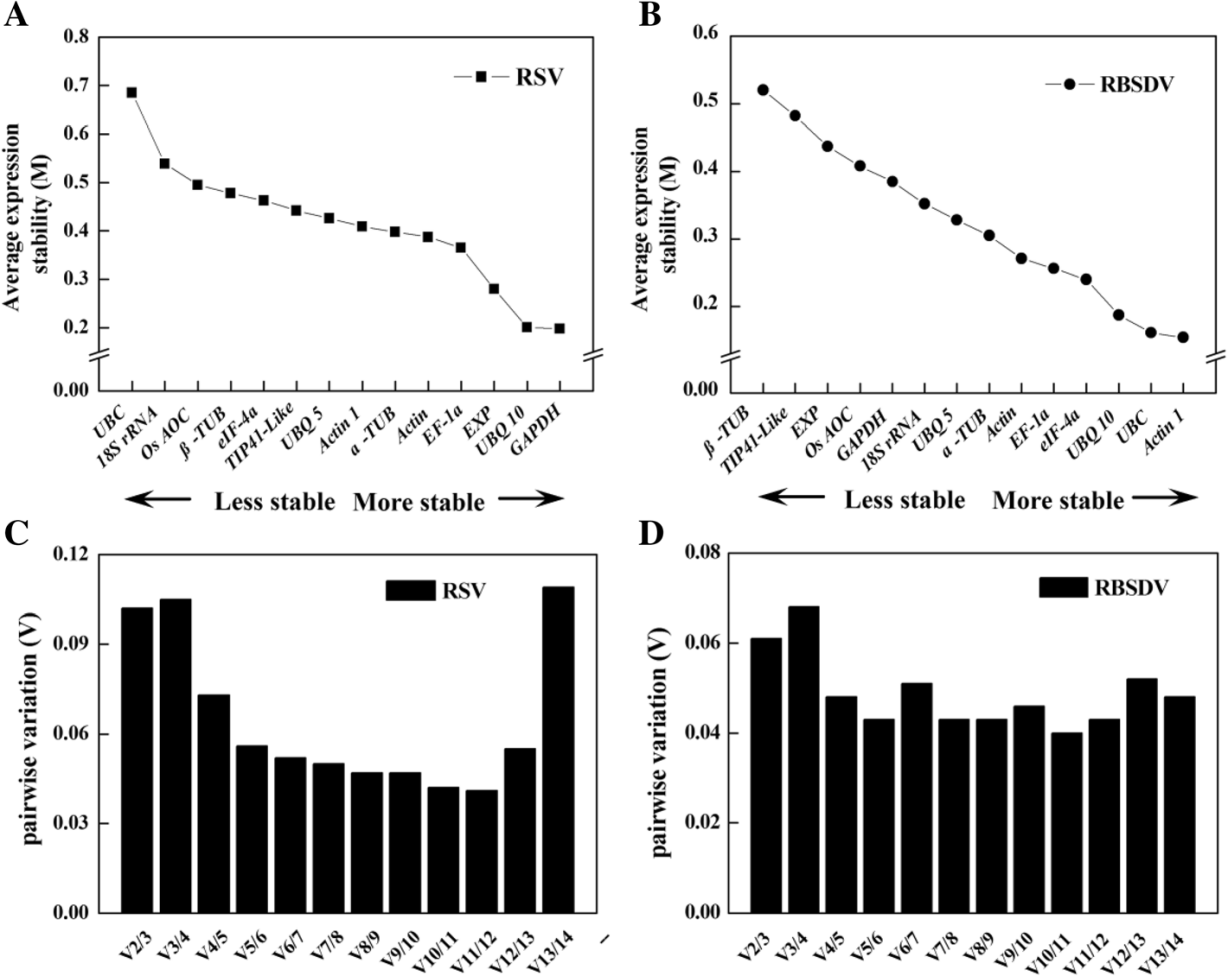
Average expression stability values (M) and pairwise variation (*V*) analyses of candidate reference genes under virus-infected conditions by geNorm. 14 candidate reference genes were amplified in cDNA samples from RSV- and RBSDV-infected seedlings. A lower M value indicated more stable expression. Mean expression stability following stepwise exclusion of the least stable gene across samples from RSV-infected treatments (**a**) or RBSDV-infected samples (**b**), respectively. The pairwise variation (*V*_n/n+1_) measured the effect of adding additional reference genes on the normalization factor (The dash line denotes 0.15 cut-off *V* value) for these treatments (**c** and **d**). Calculations were performed as described in “Materials and Methods” section

In our experimental conditions, geNorm analysis showed that *GAPDH* and *UBQ 10* were ranked as the best reference genes in RSV-infected plants, followed by *EXP*, while *UBC* and *18S rRNA* were the least stable genes (Fig. 4a). When considering RBSDV-infected treatments, the average expression stability value (M) of *Actin1* and *UBC* were two most stable genes, followed by *UBQ 10*, and those of *β-TUB* and *TIP41-like* were the highest two (Fig. 4b).

In general, it is not sufficient by using only one most stable reference gene to obtain an accurate and reliable result. Therefore, the next question is how many reference genes should be included for RT-qPCR normalization. The geNorm software also calculated the pairwise variations (*V*_*n/n+1*_) between two sequential normalization factors to determine the necessity of adding further reference gene(s). As suggested by Vandesompele et al. [28], 0.15 is a cutoff *V* value, below which the inclusion of an additional reference gene is not required. But this proposed value can not be taken as an absolute rule and might depend on the data. It is advisable to add additional reference genes to the normalization factor until the added gene has no significant effect [28, 30].

The pairwise variation analyses showed that all of *V* values were less than 0.15 in this set of samples (Fig. 4c and d). Moreover, the *V*_3/4_ values were higher than that of *V*_2/3_ and *V*_4/5_. Although *V*_4/5_ values were much lower than that of *V*_3/4_ and even lower values were obtained by adding more reference genes, considering practical applications, two reference genes was optimal in our experimental conditions.

**Fig. 5:**
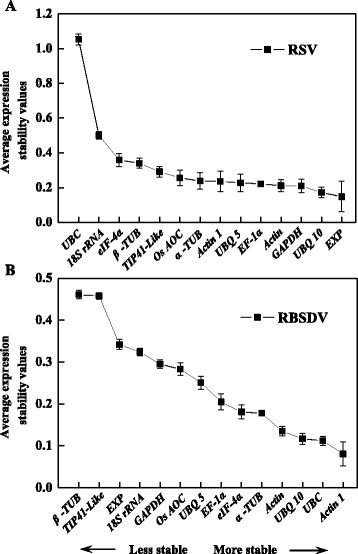
The ranking of candidate reference genes based on stability values calculated by NormFinder under virus-infected conditions. 14 candidate reference genes were amplified in cDNA samples from RSV- and RBSDV-infected seedlings. Relative quantifications of RSV-infected (**a**) or RBSDV-infected (**b**) seedlings, were performed respectively, as described in “Materials and Methods” section

Different algorithm methods sometimes may produce different results from the same data set. Hence, all of the data were reassessed by NormFinder to avoid introducing unnecessary bias. NormFinder is a mathematical model which performs separate analysis of sample subgroups. It estimates intra- and inter-group variations and combines both results into a consistent value for each investigated gene [29]. Interestingly, we found that the ranking generated by this approach (Fig. 5) was approximately similar with those determined by geNorm (Fig. 4a and b). *UBC* and *18S rRNA* were sill ranked higher than other reference genes in RSV-infected samples. In the RBSDV-infected samples, *β-TUB* and *TIP41-Like* were also ranking the highest. Although in RSV-infected samples, the rank order of *EXP* and *GAPDH* were slightly altered in NormFinder analysis in comparison with those in geNorm analyses, the stability of *UBQ 10* + *GAPDH* was high enough for reliable normalization compared to those of *UBC* and *18S rRNA*. Also, the similar result was obtained in the RBSDV-treated samples, showing that *Actin1* + *UBC* were suitable for normalization in RT-qPCR analysis.

### Comparison of single and multiple reference gene(s) in quantitative Real-time PCR normalization

**Fig. 6:**
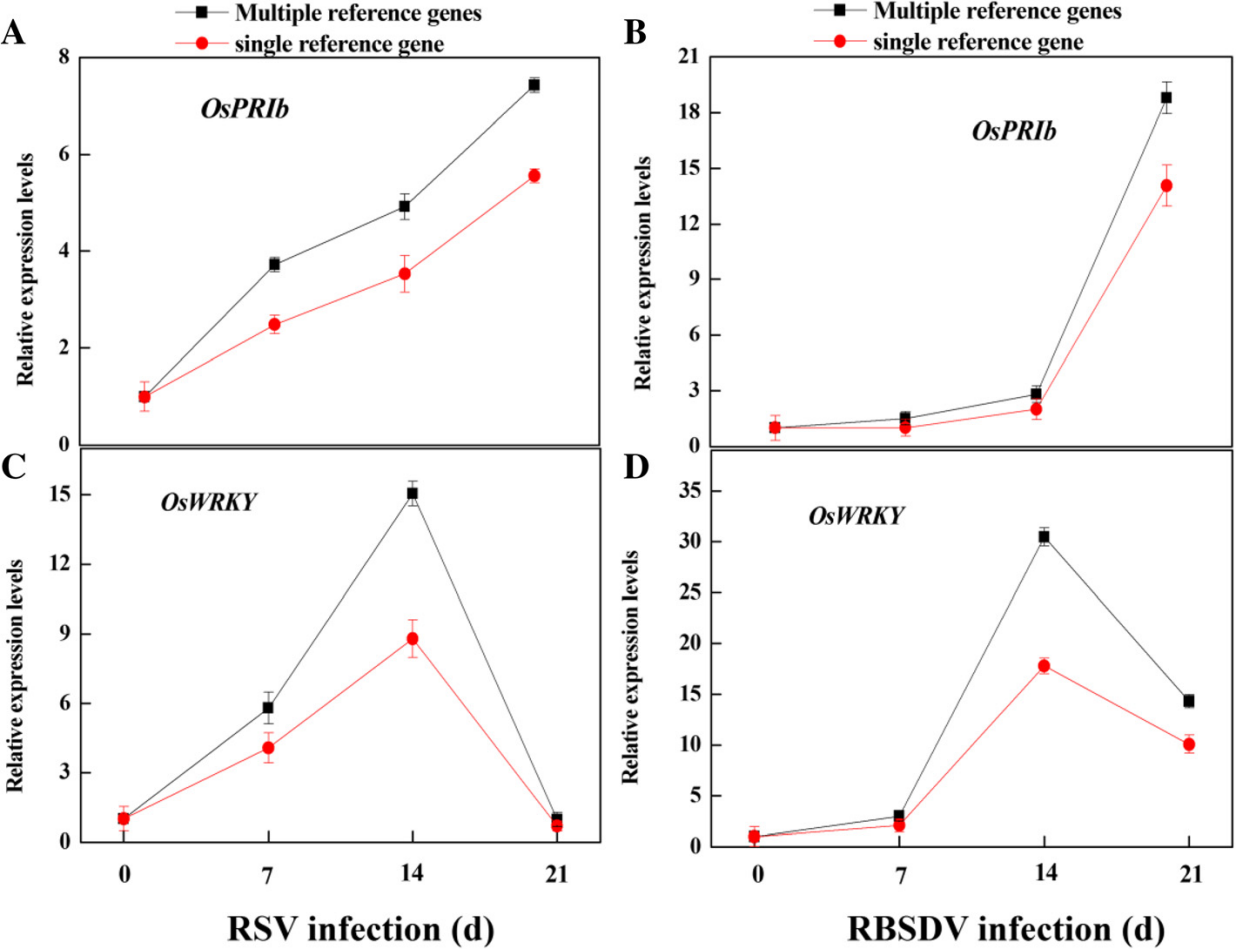
Relative expression levels of *OsPR1b* and *OsWRKY* using single or multiple reference gene(s) for normalization during RSV- (**a** and **c**) and RBSDV- (**b** and **d**) infection. 14-day-old rice seedlings were inoculated with viruliferous nymphs (RSV and RBSDV) for 3 days. Total RNA was extracted from RSV- and RBSDV-infected seedlings, respectively. *TIP41-Like* was used as a single reference gene, while *UBQ 10* + *GAPDH* and *UBC* + *Actin1* were used multiple reference genes under RSV- and RBSDV-infection plants, respectively, in our experimental conditions. Bars indicate standard error of the mean

In order to demonstrate the usefulness of the above validated reference genes in RT-qPCR, two important genes in virus resistant response, *OsPR1b* [31–33] and *OsWRKY* [34–37], were selected and applied as target genes (Fig. 6). In RSV- and RBSDV-infected samples, *UBQ 10* + *GAPDH* and *UBC* + *Actin1* were used as multiple reference genes, respectively. Meanwhile, *TIP41-Like* was used as single reference gene in our experimental conditions. As expected, in comparison with single reference gene, the normalization of *OsPR1b* using *UBQ 10* + *GAPDH* and *UBC* + *Actin1*, respectively, resulted in significant increase in transcript levels under virus infection conditions (Fig. 6a and b). The result was similar with previous studies [38, 39]. Meanwhile, the expression levels of *OsWRKY* were progressively increased in the early stage of infection and then decreased (Fig. 6c and d). By contrast, when *TIP41-Like* was used as single reference gene, the increasing transcript patterns of *OsPR1b* and *OsWRKY* were differentially lower than those normalized by multiple reference genes.

**Fig. 7:**
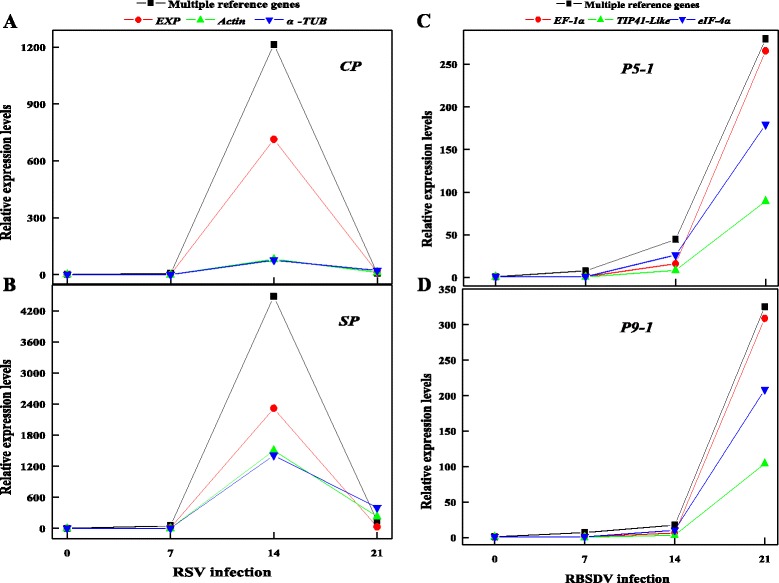
Relative expression levels of virus gene*s* (*CP* and *SP* for RSV, *P5-1* and *P9-1* for RBSDV) using single or multiple reference gene(s) for normalization during RSV- (**a** and **c**) and RBSDV- (**b** and **d**) infection. 14-day-old rice seedlings were inoculated with viruliferous nymphs (RSV and RBSDV) for 3 days. Total RNA was extracted from RSV- and RBSDV-infected seedlings, respectively. *UBQ 10* + *GAPDH* and *UBC* + *Actin1* were used multiple reference genes under RSV- and RBSDV-infection plants, respectively

We also detect the virus gene expression in plants during viral infection. According to the results, the expression patterns of the virus gene are consistent with the expression level of virus resistant gene (Fig. 7). The expression level normalized by multiple reference genes were better than those by single reference, therefore multiple reference genes were suitable for normalization in RT-qPCR analysis.

## Discussion

RSV and RBSDV are two significant rice viruses that threat rice production. To understand the mechanism of viral transmission and discover some resistance genes, a reliable quantitation method is particularly needed. RT-qPCR is still the most commonly used technique. However, an accurate and reliable gene expression analyzed by RT-qPCR highly requires stably expressed reference genes for normalization. In fact, no one gene can act as a universal reference under different experimental conditions, and the normalization of gene expression with a single reference gene can usually lead to relatively errors [28, 30]. Thus, two or more stably reference genes for normalizing are essential.

In this study, the suitable reference genes for normalizing gene expression in RSV- and RBSDV-infected rice plants were identified. We first assessed the integrity of RNA samples (Fig. 2c). Meanwhile, the specificity of the RT-qPCR primer pairs was confirmed by agarose gel electrophoresis (Fig. 2b) and melting curves analysis (Fig. 2a). The relative abundance of candidate reference genes was further identified (Fig. 3). The results of these indicated that the candidate reference genes could be used for the subsequent analyses.

To date, the commonly used methods to assess the stability of reference genes are geNorm [28], NormFinder [29] and BestKeeper [40]. As BestKeeper cannot test more than 10 candidates, it was not used in this study. By using geNorm and NormFinder, algorithms, 14 reference genes (Table 1) were evaluated. Our findings revealed that *UBQ 10* and *GAPDH* were overall the most stable genes in RSV-infected rice (Figs. 4a and 5a), and *Actin1* and *UBC* ranked the best candidate genes under RBSDV infection (Figs. 4b and 5b). Interestingly, the suitable reference genes for RSV- and RBSDV-infected rice plants were different, it is indicated that the expression of the reference genes can vary under given situations [21]. Therefore, it is vital to choose suitable reference gene(s) for normalization of gene expression. Moreno et al. [41] showed that in different *Cassava brown streak virus* (CBSV)-infected tissues, *PP2A*, *UBQ10* and *GTPb* appeared to be the most stable genes. In infected tomato plants, *GAPDH* and *UBQ* indicated as the most appropriate internal standards both in leaves and root tissues, and *Actin* was also stably expressed in the infected plants [42]. When infected with four commonly known tomato viral pathogens, *ACT*, *CAC* and *EF1-α* were considered as the most suitable reference genes in the studies of host–virus interactions [43]. Therefore, the selected multiple reference genes (*UBQ 10* + *GAPDH* for RSV, and *UBC* + *Actin1* for RBSDV) could be used for the normalization of gene expression pattern in RSV- and RBSDV-infected rice plants.

Normally, *EF1-α* and *18S rRNA* were used as the reference genes in RT-qPCR experiments. Previous results showed that *EF1-α* was expressed stably in potato during biotic and abiotic stress [44], and *18S rRNA* was identified to be suitable for normalisation in *Barley yellow dwarf virus*-infected cereals [45]. In our study, the performance of above two genes was dissatisfactory. These were similar to the earlier studies in *Cicer arietinum* and virus-infected tomato, showing the considerable alterations in the transcript levels of *EF1-α* and *18S rRNA* [42, 46]. Thus, the two traditional reference genes might not be the optimal choices for quantitating transcript level in rice during RSV- and RBSDV-infection. Meanwhile, *TUB* was widely used as reference gene for gene expression analysis. However, due to the potential regulation in various physiological states, the suitability as internal control has been questioned [45, 47]. In our experimental conditions, *α-TUB* and *β-TUB* (in particularly) were not stable either, especially in RBSDV-infected plants*.* Taken together, our data demonstrated that *α-TUB* and *β-TUB* were unsuitable for the normalization of gene expression levels in viral infected rice.

To further investigate the suitability of the selected reference genes, the expression levels of two virus resistant response genes, the gene expression of *OsPR1b* and *OsWRKY*, was compared by using single and multiple reference gene(s) for normalization. In the earlier studies [38, 39], for example, the expression of *OsPR1b* gene was strongly induced by virus infection*.* Our results further showed that when using *TIP41-Like* gene for normalization, the induction patterns of *OsPR1b* and *OsWRKY* transcripts were not stronger than those using two reference genes (Fig. 6). Thus, the application of multiple reference genes is a better choice for gene expression analysis under virus infection situation.

In conclusion, we evaluated 14 candidate reference genes, and identified *UBQ 10* + *GAPDH* and *UBC* + *Actin1* as the most stably expressed reference genes in RSV- and RBSDV-infected rice, respectively. These genes will enable more accurate and reliable normalization of RT-qPCR analysis for gene expression studies to get insight into complex regulatory networks and will most probably lead to the identification of genes relevant to new biological processes, in RSV- and RBSDV-infected plants.

## Methods

### Plant materials, virus isolates and inoculation assay

Rice (*Oryza sativa* L. cv. Nipponbare) seeds were used throughout the experiments. After the disinfection with 0.1 % HgCl_2_ for 1 h and thorough washing with RO (reverse-osmosis) water, seeds were soaked overnight in RO water and incubation at 25 °C for 1 day. Seedlings were then grown on plastic chambers using Kimura B nutrient solution [48], with a 14/10 h (day/night) regimes at 28 ± 1 °C.

Rice plants infected with RSV and RBSDV, were collected from Jianhu county, Jiangsu Province in July 2014. Young instar nymphs of SBPHs were fed RSV- and RBSDV-infected rice plants for 2 days to acquire the virus, respectively. The virus was maintained by SBPHs in an insect-rearing room at a temperature of 25 °C. Virus-free SBPHs were also used for the control inoculation. Viruliferous or virus-free SBPHs were reared on rice seedlings (*Oryza sativa* L. cv. Wuyujing No. 3) in glass vessels at 22 °C under alternating photoperiods of 14 h of light and 10 h of dark. 14-day-old seedlings were inoculated with 10 viruliferous nymphs per plant and were kept in a growth chamber. After the incubation for 3 days, planthoppers were removed and plants were transferred to field. Virus-free SBPHs were also used for control inoculation. Leaf tissues were collected after 7, 14 and 21 days of virus treatment and immediately frozen in liquid nitrogen and stored at −80 °C until further analyses.

According to previous report [49], RT-PCR was carried out to detect RSV and RBSDV in rice plants. Primer RSRB-R (5′-CCYATCACAAASAAATMAAAAT-3′) paired with primer RSV-F (5′-AGATCCAGAGAGAGTCACGGAAG-3′) was used to amplify a specific 1114 bp fragment for detection of RSV. Primers RSRB-R and RBSDV-F (5′-GTTCAAAGACAATACACTCAAAA-3′) were used to amplify a 414 bp product, which was specific for RBSDV.

### Preparation and quantification of DNA-free total RNA

Total RNA was extracted by Trizol reagent (Invitrogen, Gaithersburg, MD, USA) according to the manufacturer’s instructions. The RNA was dissolved in DNase-treated distilled water. The concentration and quality of isolated RNA were determined using the NanoDrop 2000 spectrophotometer (Thermo Fisher Scientific, Wilmington, DE, USA). Only the RNA samples with 260/280 ratio (an indication of protein contamination) between 1.9 and 2.1 and 260/230 ratio (an indication of reagent contamination) greater than 2.0, were used for the analyses [50, 51]. The integrity of RNA samples was assessed by agarose gel electrophoresis. All RNA samples were adjusted to the same concentration and measured again to homogenize RNA for the subsequent experiments.

### Reverse transcription and quantitative Real-time PCR assay

cDNA was synthesized from 2 μg of total RNA using an oligo (dT) primer and M-MLV reverse transcriptase (BioTeke, Beijing, China). RT-qPCR was performed using the SsoFast^TM^ Eva Green® Supermix (Bio-Rad) with the Bio-Rad iQ5 RT-qPCR system. Combined with all internal control genes used recently [25, 27, 28], we further selected 14 genes as the candidate reference genes in this study (Table 1). Additionally, the specific primers 5′-ACGCCTTCACGGTCCATAC-3′ and 5′-AAACAGAAAGAAACAGAGGGAGTAC-3′ were used for *OsPR1b* (AK107926); 5′-TCAGTGGAGAAGCGGGTGGTG-3′ and 5′-GGGTGGTTGTGCTCGAAGGAG-3′ were used for *OsWRKY* (EF143611). The efficiency and specificity of all the primers were checked by melting curve analysis, similar to previous report [51].

Using the RSV *CP*, *SP* gene and RBSDV *P5-1, P9-1* gene*,* the dynamics of viral infection in rice plants was measured by reverse-transcription real-time PCR. The primer and products were shown in Table 2.

**Table 2:** Real-time PCR primers of virus genes (RSV and RBSDV) used in this study

Gene name	Primer sequence (5′ → 3′)	Amplicon length (bp)	Target genes
*CP-F*	TGCAGAAGGCAATCAATGACAT	150	RSV NCP
*CP-R*	TGTCACCACCTTTGTCCTCAA		
*SP-F*	CCTGTTAGGAGGTGAAGATGATGA	180	RSV SP
*SP-R*	GCTCTCAGCCTTAGCCATCTTG		
*P5-1-F*	GTTTACGGTGGTGCAATTTTCA	150	RBSDV P5-1
*P5-1-R*	AGGCTTTCCTTCACTAACTTCTGACT		
*P9-1-F*	TGGTGCTTCTCGTCAAACTGTCT	100	RBSDV P9-1
*P9-1-R*	GCCAACAATTCGTGTCCTGAA		

Each quantitative Real-time PCR was performed using 0.5 μL cDNA, 10 μL of SsoFast^TM^ Eva Green® Supermix and 0.2 μM forward and reverse primers were used in a total volume of 20 μL. All tubes were subjected to denaturation for 10 min at 95 °C, followed by 40 cycles of 95 °C for 10 s, and 56 °C for 20 s. SYBR Green absorbance was detected at 56 °C. All reactions were conducted in triplicate. Amplicon dissociation curves (melting curves), were recorded after cycle 40 by heating from 60 °C to 95 °C at a ramp speed of 1.9 °C ⁄min.

### Data processing

The expression stability of the candidate reference genes were analyzed by using geNorm [28] and NormFinder [29]. Expression levels were assessed based on the number of amplification cycles needed to reach a specific threshold (cycle threshold; Ct) in the exponential phase of RT-qPCR. For both programs, raw Ct values of each gene were converted into relative quantities before inputting into software. The relative expression levels of corresponding genes were calculated relative to the maximum abundance in different samples. The highest relative expression for each gene was set to 1.0. The geNorm algorithm [28] derives a stability measure (M). Via a stepwise exclusion of the least stable gene, it creates a stability ranking. It also estimates the number of genes required to calculate a robust normalization factors, and performs a stepwise analysis (more stable to less stable genes) to calculate the pairwise variation *(V*_*n/n+1*_*)* between two sequential normalization factors containing an increasing number of genes. NormFinder algorithm [29] used an ANOVA-based model to estimate intra- and inter-group variation. It combines these results to provide a direct measurement of the variation in the expression for each gene [52]. Statistical significance of Ct differences between treatments was calculated by the Mann–Whitney *t* test using the GraphPad Prism 5 software.

## Additional file

Additional file 1: Figure S1. Relative expression levels of *OsPR1b* and *OsWRKY* using single or multiple reference gene(s) for normalization during RSV- (A and C) and RBSDV- (B and D) infection. 14-day-old rice seedlings were inoculated with viruliferous nymphs (RSV and RBSDV) for 3 days. Total RNA was extracted from RSV- and RBSDV-infected seedlings, respectively. In our experimental conditions, *UBQ 10* + *GAPDH* and *UBC* + *Actin1* were used multiple reference genes under RSV- and RBSDV-infection plants. (DOC 303 kb)

## References

[1] Hibino H (1996). Biology and epidemiology of rice viruses. Annu Rev Phytopathol.

[2] Kuribayashi K (1931). On the relationship between rice stripe disease and Delphacodes striatella Fallen. J Plant Prot.

[3] Shikata E, Kitagawa Y (1977). Rice black-streaked dwarf virus: its properties, morphology and intracellular localization. Virology.

[4] Falk BW, Tsai JH (1998). Biology and molecular biology of viruses in the genus *Tenuivirus*. Annu Rev Phytopathol.

[5] Kiso A, Yamamoto T (1973). Infection and symptom in rice stripe disease with special reference to disease-specific protein other than virus. Rev Plant Prot Res.

[6] Shinkai A (1962). Studies on insect transmission of rice virus diseases in Japan. Bull Nat Inst Agric Sci Ser C.

[7] Iida TT, Shinkai A. Transmission of dwarf, yellow dwarf, stripe and black-streaked dwarf. In: RF Chandler, editor. The virus diseases of the rice plant. International Rice Research Institute, the Johns Hopkins Press: Baltimore, MD; 1969. p. 125–129.

[8] Sun DZ, Jiang L (2006). Research on the inheritance and breeding of rice stripe resistance. Chin Agric Sci Bull.

[9] Fang S, Yu J, Feng J, Han C, Li D, Liu Y (2001). Identification of rice black-streaked dwarf fijivirus in maize with rough dwarf disease in China. Arch Virol.

[10] Isogai M, Uyeda I, Lee BC (1998). Detection and assignment of proteins encoded by rice black streaked dwarf fijivirus S7, S8, S9 and S10. J Gen Virol.

[11] Dong GK, Wang EG, Luo GL, Lin LW, Guan MP, Zhang ZD (1999). Occurrence of rice black-streaked dwarf disease in late season hybrid rice and its control strategy. Acta Agric Zhejiangensis.

[12] Gachon C, Mingam A, Charrier B (2004). Real-time PCR: what relevance to plant studies?. J Exp Bot.

[13] Bustin SA, Benes V, Nolan T, Pfaffl MW (2005). Quantitative real-time RT-PCR–a perspective. J Mol Endocrinol.

[14] Dekkers BJW, Willems L, Bassel GW, van Bolderen-Veldkamp RP, Ligterink W, Hilhorst HWM (2012). Identification of reference genes for RT-qPCR expression analysis in Arabidopsis and tomato seeds. Plant Cell Physiol.

[15] Bustin SA, Nolan T (2004). Pitfalls of quantitative real-time reverse-transcription polymerase chain reaction. J Biomol Tech.

[16] Huggett J, Dheda K, Bustin S, Zumla A (2005). Real-time RT-PCR normalisation; strategies and considerations. Genes Immun.

[17] Skern R, Frost P, Nilsen F (2005). Relative transcript quantification by quantitative PCR: roughly right or precisely wrong?. BMC Mol Biol.

[18] Czechowski T, Stitt M, Altmann T, Udvardi MK, Scheible WR (2005). Genome-wide identification and testing of superior reference genes for transcript normalization in Arabidopsis. Plant Physiol.

[19] Lee PD, Sladek R, Greenwood CMT, Hudson TJ (2002). Control genes and variability: absence of ubiquitous reference transcripts in diverse mammalian expression studies. Genome Res.

[20] Radonic A, Thulke S, Mackay IM, Landt O, Siegert W, Nitsche A (2004). Guideline to reference gene selection for quantitative real-time PCR. Biochem Biophys Res Commun.

[21] Thellin O, Zorzi W, Lakaye B, De Borman B, Coumans B, Hennen G (1999). Housekeeping genes as internal standards: use and limits. J Biotechnol.

[22] Gutierrez L, Mauriat M, Guénin S, Pelloux J, Lefebvre JF, Louvet R (2008). The lack of a systematic validation of reference genes: a serious pitfall undervalued in reverse transcription-polymerase chain reaction (RT-PCR) analysis in plants. Plant Biotechnol J.

[23] Seddas P, Boissinot S, Strub JM, Van Dorsselaer A, Van Regenmortel MH, Pattus F (2004). Rack-1, GAPDH3, and actin: proteins of *Myzus persicae* potentially involved in the transcytosis of beet western yellows virus particles in the aphid. Virology.

[24] Tamborindeguy C, Monsion B, Brault V, Hunnicutt L, Ju HJ, Nakabachi A (2010). genomic analysis of transcytosis in the pea aphid, *Acyrthosiphon pisum*, a mechanism involved in virus transmission. Insect Mol Biol.

[25] Brenndörfer M, Boshart M (2010). Selection of reference genes for mRNA quantification in Trypanosoma brucei. Mol Biochem Parasitol.

[26] Lord JC, Hartzer K, Toutges M, Oppert B (2010). Evaluation of quantitative PCR reference genes for gene expression studies in Tribolium castaneum after fungal challenge. J Microbiol Methods.

[27] Radonic A, Thulke S, Bae HG, Muller M, Siegert W, Nitsche A (2006). Reference gene selection for quantitative real-time PCR analysis in virus infected cells: SARS corona virus, Yellow fever virus, Human Herpesvirus-6, Camelpox virus and Cytomegalo virus infections. Virol J.

[28] Vandesompele J, De Preter K, Pattyn F, Poppe B, Van Roy N, De Paepe A (2002). Accurate normalization of real-time quantitative RT-PCR data by geometric averaging of multiple internal control genes. Genome Biol.

[29] Andersen CL, Jensen JL, Ørntoft TF (2004). Normalization of real-time quantitative reverse transcription-PCR data: a model-based variance estimation approach to identify genes suited for normalization, applied to bladder and colon cancer data sets. Cancer Res.

[30] Gutierrez L, Mauriat M, Pelloux J, Bellini C, Van Wuytswinkel O (2008). Towards a systematic validation of references in real-time RT-PCR. Plant Cell.

[31] Hao ZN, Wang LP, He YP, Liang JG, Tao RX (2011). Expression of defense genes and activities of antioxidant enzymes in rice resistance to rice stripe virus and small brown planthopper. Plant Physiol Biochem.

[32] Satoh K, Kondoh H, De Leon T, Macalalad RJA, Cabunagan RC, Cabauatan PC (2013). Gene expression responses to Rice *tungro spherical virus* in susceptible and resistant near-isogenic rice plants. Virus Res.

[33] Lee KJ, Kim K. The rice serine/threonine protein kinase OsPBL1 (ORYZA SATIVA ARABIDOPSIS PBS1- LIKE 1) is potentially involved in resistance to rice stripe disease. Plant Growth Regul. 2015, 1–9. doi: 10.1007/s10725-015-0036-z.

[34] Qiu D, Xiao J, Ding X, Xiong M, Cai M, Cao Y (2007). OsWRKY13 mediates rice disease resistance by regulating defense-related genes in salicylate- and jasmonate-dependent signaling. Mol Plant Microbe Interact.

[35] Bai FW, Yan J, Qu ZC, Zhang HW, Xu J, Ye MM (2002). Phylogenetic analysis reveals that a dwarfing disease on different cereal crops in China is due to rice black streaked dwarf virus (RBSDV). Virus Genes.

[36] Catoni M, Miozzi L, Fiorilli V, Lanfranco L, Accotto GP (2009). Comparative analysis of expression profiles in shoots and roots of tomato systemically infected by *Tomato spotted wilt virus* reveals organ-specific transcriptional responses. Mol Plant Microbe Interact.

[37] Tao Z, Liu H, Qiu D, Zhou Y, Li X, Xu C (2009). A pair of allelic *WRKY* genes play opposite roles in rice–bacteria interactions. Plant Physiol.

[38] Satoh K, Kondoh H, Sasaya T, Shimizu T, Choi IR, Omura T (2010). Selective modification of rice (*Oryza sativa*) gene expression by rice stripe virus infection. J Gen Virol.

[39] Zheng WJ, Ma L, Zhao JM, Li ZQ, Sun FY, Lu XU (2013). Comparative transcriptome analysis of two rice varieties in response to rice stripe virus and small brown planthoppers during early interaction. PLoS One.

[40] Pfaffl MW, Tichopad A, Prgomet C, Neuvians TP (2004). Determination of stable housekeeping genes, differentially regulated target genes and sample integrity: BestKeeper Excel-based tool using pair-wise correlations. Biotechnol Lett.

[41] Moreno I, Gruissem W, Vanderschuren H (2011). Reference genes for reliable potyvirus quantitation in cassava and analysis of *Cassava brown streak virus* load in host varieties. J Virol Methods.

[42] Mascia T, Santovito E, Gallitelli D, Cillo F (2010). Evaluation of reference genes for quantitative reverse-transcription polymerase chain reaction normalization in infected tomato plants. Mol Plant Pathol.

[43] Wieczorek P, Wrzesińska B, Obrępalska-Stęplowska A (2013). Assessment of reference gene stability influenced by extremely divergent disease symptoms in *Solanum lycopersicum* L. J Virol Methods.

[44] Wan H, Zhao Z, Qian C, Sui Y, Malik AA, Chen J (2010). Selection of appropriate reference genes for gene expression studies by quantitative real-time polymerase chain reaction in cucumber. Anal Biochem.

[45] Jarošová J, Kundu J (2010). Validation of reference genes as internal control for studying viral infections in cereals by quantitative real-time RT-PCR. BMC Plant Biol.

[46] Castro P, Roman B, Rubio J, Die JV (2012). Selection of reference genes for expression studies in *Cicer arietinum* L.: analysis of *cyp81E3* gene expression against *Ascochyta rabiei*. Mol Breed.

[47] Mafra V, Kubo KS, Alves-Ferreira M, Ribeiro-Alves M, Stuart RM, Boava LP (2012). Reference genes for accurate transcript normalization in citrus genotypes under different experimental conditions. PLoS One.

[48] Ma JF, Goto S, Tamai K, Ichii M (2011). Role of root hairs and lateral roots in silicon uptake by rice. Plant Physiol.

[49] Li S, Wang X, Xu JX, Ji YH, Zhou YJ (2015). A simplified method for simultaneous detection of Rice stripe virus and Rice black-streaked dwarf virus in insect vector. J Virol Methods.

[50] Nolan T, Hands RE, Bustin SA (2006). Quantification of mRNA using real-time RT-PCR. Nat Protoc.

[51] Kumar K, Muthamilarasan M, Prasad M (2013). Reference genes for quantitative real-time PCR analysis in the model plant foxtail millet (*Setaria italica* L.) subjected to abiotic stress conditions. Plant Cell Tiss Org.

[52] Chi XY, Hu RB, Yang QL, Zhang XW, Pan LJ, Chen N (2012). Validation of reference genes for gene expression studies in peanut by quantitative real-time RT-PCR. Mol Genet Genomics.

