# Supplementary material for: Assessment of reference gene stability in Rice stripe virus and Rice black streaked dwarf virus infection rice by quantitative Real-time PCR
Source: Virol J. 2015 Oct 24;12:175. doi: 10.1186/s12985-015-0405-2 (PMC4619528; doi:10.1186/s12985-015-0405-2)
Supplement: Additional file 1: Figure S1. — Relative expression levels of OsPR1b and OsWRKY using single or multiple reference gene(s) for normalization during RSV- (A and C) and RBSDV- (B and D) infection. 14-day-old rice seedlings were inoculated with viruliferous nymphs (RSV and RBSDV) for 3 days. Total RNA was extracted from RSV- and RBSDV-infected seedlings, respectively. In our experimental conditions, UBQ 10 + GAPDH and UBC + Actin1 were used multiple reference genes under RSV- and RBSDV-infection plants. (DOC 303 kb) [file 12985_2015_405_MOESM1_ESM.doc]

**Additional file 1**


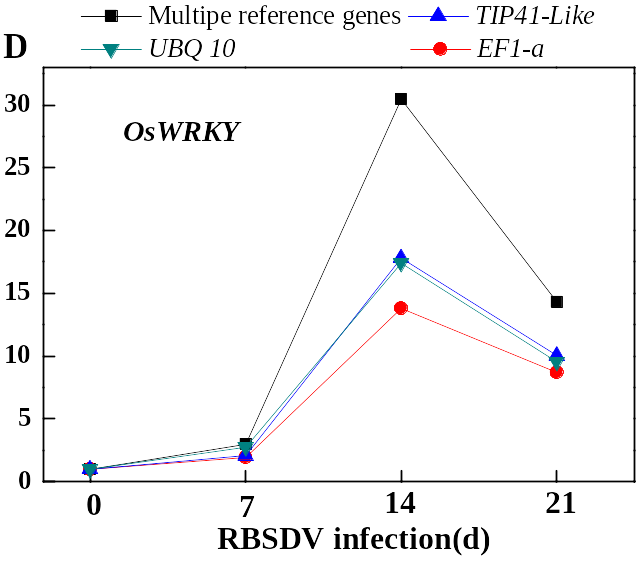

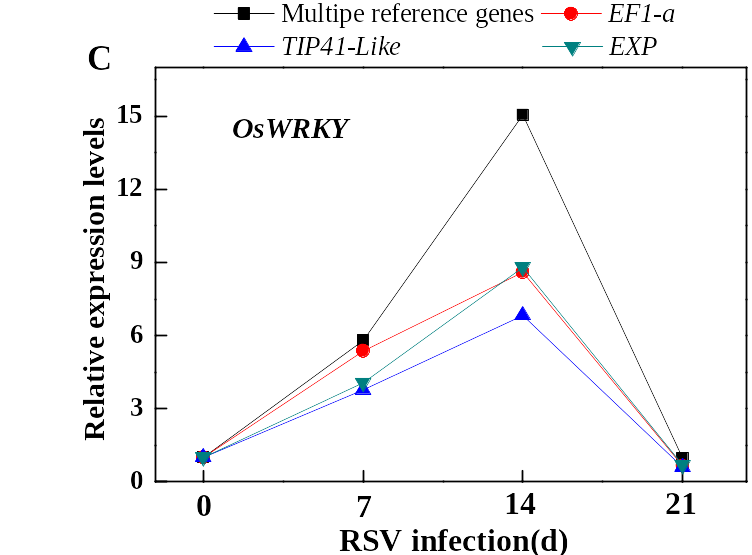

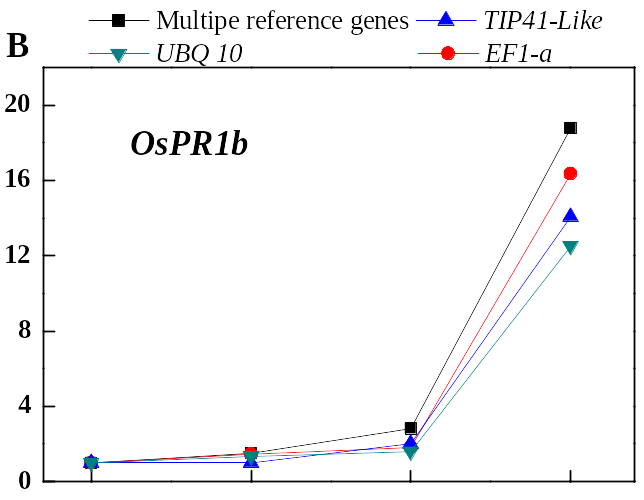

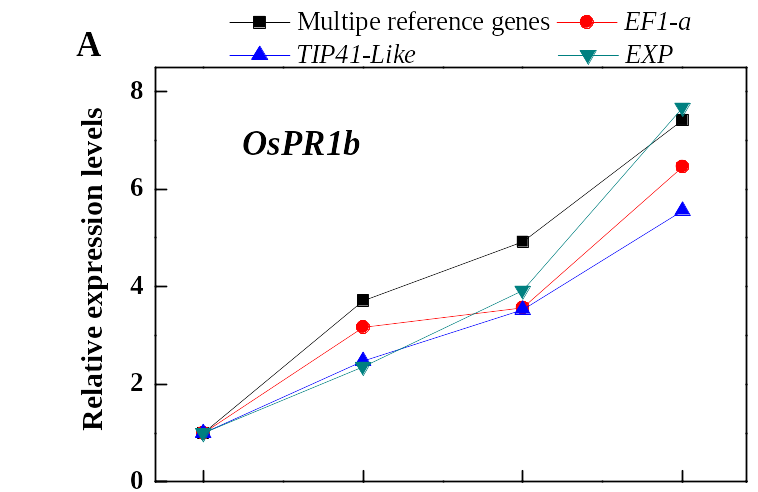


**Figure S1.** Relative expression levels of *OsPR1b* and *OsWRKY* using single or multiple reference gene(s) for normalization during RSV- (**A** and **C**) and RBSDV- (**B** and **D**) infection. 14-day-old rice seedlings were inoculated with viruliferous nymphs (RSV and RBSDV) for 3 days. Total RNA was extracted from RSV- and RBSDV-infected seedlings, respectively. In our experimental conditions, *UBQ 10* + *GAPDH* and *UBC* + *Actin1* were used multiple reference genes under RSV- and RBSDV-infection plants.
